# Behavioral intervention with task repetition compared to pharmacological intervention with SSRI for enhancement of cognitive control in emotional and non-emotional settings

**DOI:** 10.1007/s00213-025-06835-6

**Published:** 2025-06-19

**Authors:** Myrto Sklivanioti Greenfield, Yanlu Wang, Lina Martinsson, Tie-Qiang Li, Mussie Msghina

**Affiliations:** 1 Department of Clinical Neuroscience (CNS), Karolinska Institute, Stockholm, Sweden; 2 Department of Nuclear Medicine Medical Physics, Karolinska University Hospital, Stockholm, Sweden; 3 Department of Oncology-Pathology, Karolinska Institute, Stockholm, Sweden; 4 Department of Clinical Science, Intervention, and Technology, Karolinska Institute, Stockholm, Sweden; 5 Department of Psychiatry, School of Medicine, Faculty of Medicine and Health, Örebro University, Örebro, Sweden

**Keywords:** PFC, Cognitive control, Emotion regulation, Training, Escitalopram, FNIRS, EDA

## Abstract

**Rationale:**

Cognitive control is crucial for optimal daily functioning and for emotional well-being. Cognitive control has been shown to be modified by experimental manipulations under widely differing experimental conditions, including cognitive training, and pharmacological intervention mainly probing catecholaminergic systems with little focus on the serotonergic system.

**Objectives:**

To explore the role of serotonin on cognitive control in emotional and non-emotional settings.

**Methods:**

Behavioral, electrodermal and prefrontal activity measures were evaluated to compare the effects of single-session task repetition and single-dose serotonergic intervention with escitalopram on cognitive control in healthy participants, using cognitive and emotional Stroop tasks.

**Results:**

For cognitive Stroop, task repetition improved performance both ‘on-line’ within an ongoing task and ‘off-line’ after a four-hour delay, and escitalopram had no additional effects on this. In emotional Stroop, escitalopram enhanced the practice-related performance gain, starting from the second stimulus of each block. Compared to placebo, escitalopram also significantly reduced overall rate of premature responses. Regarding brain activation, escitalopram significantly reduced prefrontal activity during cognitive and even more so during emotional Stroop task. Lastly, electrodermal response showed significant habituation during cognitive but not emotional Stroop, in an effect that was not significantly modified by escitalopram.

**Conclusions:**

Cognitive control in emotional and non-emotional settings may respond differently to behavioral and pharmacological manipulations. Escitalopram may selectively improve cognitive control in an emotional setting compared to cognitive control in non-emotional settings.

**Supplementary Information:**

The online version contains supplementary material available at 10.1007/s00213-025-06835-6.

## Introduction

Goal-directed behavior requires ability to focus on relevant information for a task at hand and to effectively ignore irrelevant sensory, cognitive, and affective distractions. Cognitive control is central for optimal daily functioning, and its dysregulation is associated with suboptimal performance in the non-clinical population (Feldman Barrett et al. 2004; Gray 2004) and in considerable clinical impairment in individuals with psychiatric morbidity. Both performance at the behavioral level and brain activity at the cortical level have been shown to be altered during different measures of cognitive control in a wide spectrum of psychiatric disorders including psychosis (Weiss et al. 2003), depression (Wagner et al. 2006) and anxiety disorders (Nakao et al. 2005). Cognitive control and ways to improve it have thus become the focus of much multidisciplinary research, and in the laboratory setting the Stroop task and similar cognitive paradigms are routinely used to this end.

The Stroop task has been widely used to study cognitive control and to evaluate negative effects of different kinds of interference on optimal performance (McKenna and Sharma 2004; Rahm et al. 2013). In the prototypical Stroop design, where one aspect of the stimulus (the color of the text) has to be attended to, while the lexical aspect is to be ignored, accuracy and processing speed are compromised by interference created by the incongruency between the meaning of the word and the color of the text (Stroop 1935; MacLeod 1991). A modified version of this paradigm, the matching Stroop Task, was developed by Zysset and colleagues (Zysset et al. 2001) to account for the fact that in the classical Stroop task, response selection and interference suppression could affect each other if they are presented in the same modality, but can be better segregated if interference occurs at a more abstract level. Cognitive control in the real world does not occur in isolation, but more often than not in the presence of pertinent emotional context, and it has been shown in numerous studies that emotional and cognitive processes reciprocally influence each other during cognitive control tasks (Gray 2004; Mikels and Reuter-Lorenz 2019). Emotion is known to provide modulatory bias for information processing and compete for computational resource with task-relevant processes (Bush et al. 2000). The emotional valence of a task-irrelevant event can thus be a source of considerable interference, affecting accuracy and reaction time in another version of the Stroop task – the emotional Stroop task (Sharma and McKenna 2001, Ben-Haim et al. 2016). In the emotional Stroop task, the interference comes therefore not from conflict between the meaning of the word and the color of the text, but from emotionally charged words with negative valence (e.g., MURDER) compared to emotionally neutral words (e.g., TABLE) (Bush et al. 2000).

Numerous studies have investigated immediate and delayed effects of single session physical exercise (Chang et al. 2012; Pontifex et al. 2013), single session strategy training (Gonthier et al. 2016) and practice (Stroop 1935; MacLeod 1991; Davidson et al. 2003) and single dose pharmacological manipulations (Ilieva et al. 2015; Marraccini et al. 2016; Roberts et al. 2020a, b) on measures of cognitive control, as well as cumulative effect of their long-term administration (Neudecker et al. 2019; Sun et al. 2022). Furthermore, cognitive control in emotional and non-emotional settings are thought to represent different processes and to require different competencies (Joormann et al. 2011; Schweizer et al. 2011; Schweizer et al. 2013), and it is not well understood if different strategies would improve performance in both contexts to a similar extent or if practice in one domain would translate to improvement in the other domain. It has been generally thought that the effect of practice and task repetition in cognitive tasks is short-lived and not generalizable (Jaeggi et al. 2008; Shipstead et al. 2012; Anguera et al. 2013; Schwaighofer et al. 2015; Melby-Lervåg et al. 2016), but there is also evidence indicating otherwise. Schweizer et al. (2011), for example, found that working memory practice in the n-back task improved performance in the Stroop task and that practice with an emotional n-back task led to transferable improvement in an emotional Stroop task. Importantly, practice-mediated performance improvement can manifest itself ‘on-line’, i.e., in real-time improvement during an ongoing task or ‘off-line’, i.e., as delayed improvement outside the time-frame of an ongoing task (Stagg 2021).

Single-dose and cumulative effects of pharmacological interventions have helped elucidate the cognitive roles of dopamine, noradrenaline and acetylcholine, but this has been done to a lesser extent for serotonin (Cools and Arnsten 2022). The prefrontal cortex (PFC), particularly its medial aspects, and the dorsal raphe nuclei are reciprocally interconnected and regulate the function of each other (Adell et al. 2010). Excitatory glutamatergic and inhibitory GABAergic neurons in the PFC express inhibitory (mainly 5-HT1A) and excitatory (mainly 5-HT2A) serotonergic receptors that are differentially distributed in different regions and cortical layers (Puig and Gulledge 2011). The local and global influence of the serotonergic system on PFC function thus depends on the fine balance of the action of the transmitter on its numerous receptors. The abundant anatomical and functional interconnection between PFC and raphe nuclei nevertheless suggests that serotonin likely plays important roles in cognitive control, as has indeed been shown in previous studies (Cools et al. 2008; Harmer 2008; Zimmer et al. 2016; Cools and Arnsten 2022), although it is not fully understood which aspects of cognitive control and under what conditions this is. Reduction of serotonergic transmission via acute tryptophan depletion has been found to improve performance in the Stroop task in some (Schmitt et al. 2000), but not other studies (Gallagher et al. 2003). Moreover, both human and animal studies indicate that increased serotonergic transmission constrains impulsive choice and enables waiting (Robbins and Crockett 2010; Ye et al. 2014; Skandali et al. 2018), decreases response to aversive emotional stimuli (Robbins and Crockett 2010), reduces vigilance (Ramaekers et al. 1995, O'Hanlon et al. 1998; Schmitt et al. 2002) and enhances reversal learning and reduces perseverative behaviour (Clarke et al. 2004; Clarke et al. 2005; Walker et al. 2008; Brown et al. 2012; Rygula et al. 2014; Kanen et al. 2019; Roberts et al. 2020b, a), processes that all are directly or indirectly associated with cognitive control.

The serotonergic system is the target of most antidepressants and theories about its role in mood disorders have been influential, although hotly debated (Moncrieff et al. 2022). Serotonin is believed to exert various effects on emotional processes, depending on the specific brain regions it influences. Serotonergic modulation influences subcortical areas by affecting motivational processes (Cools et al. 2008), while its effects on cortical regions support emotion regulation (Cools et al. 2008) and cognitive flexibility (Carhart-Harris and Nutt 2017). Serotonergic manipulations modify the effects of anxiety-related aversive stimuli and prediction of punishment without affecting reward prediction per se (Cools et al. 2008; Lewis et al. 2021) and alter the interference of negative emotional stimuli on cognitive processes (Murphy et al. 2002; Evers et al. 2005; Hornboll et al. 2018, Sklivanioti Greenfield et al. 2023). SSRI may increase sensitivity to emotionally salient negative feedback (Chamberlain et al. 2006). Relevant to this is also the effect of serotonin on emotional bias in modulating learning (Crockett et al. 2009, Dayan and Huys 2009, Cools et al. 2011, Crockett and Cools 2015, Meyniel et al. 2016, Cools and Arnsten 2022).

Clinical studies with antidepressants suggest a significant role for serotonin in depression and anxiety disorders. While clinical improvements typically require several weeks of repeated administration, measurable changes in emotional processing and brain activity can be observed within hours of the first dose (Murphy et al. 2002, Harmer et al. 2003, Bhagwagar et al. 2004, Kemp and Nathan 2004, Del-Ben et al. 2005, Takahashi et al. 2005, Anderson et al. 2007, Browning et al. 2007, Grillon et al. 2007, Bigos et al. 2008, Brühl et al. 2010, Lochner et al. 2012, Grady et al. 2013, Outhred et al. 2014, Ma et al. 2015, Outhred et al. 2015, Selvaraj et al. 2017, Skandali et al. 2018). Lastly, clinical observations provide compelling evidence of serotonin's involvement in'hot'cognition. For example, findings such as that mild serotonin syndrome has been associated with heightened anxiety and increased hypervigilance (Scotton et al. 2019) and that psychedelic agents are known to evoke profound emotional experiences, often enhancing the sense of meaning and intensifying the perception of salience (Halberstadt and Nichols 2010; Carhart-Harris 2019), underscore serotonin's critical role in emotion-laden cognitive processes.

The downstream effect of the enhancement of serotonergic transmission with selective serotonin reuptake inhibitors (SSRI) is thought to implicate phasic and tonic aspects of the serotonergic transmission and different receptors, not to mention other neurotransmitter systems (Schmitt et al. 2002; Roberts et al. 2020b, a). SSRI block the serotonin transporter (SERT), and this way increase serotonin levels both in the synaptic and extra-synaptic space (Sánchez et al. 2003; Hensler 2010).

The aim of the present study was to study the effect of serotonergic manipulation on cognitive control under ‘cold’ and ‘hot’ conditions (Ochsner and Gross 2005), i.e. in emotional and non-emotional settings using cognitive and emotional Stroop tasks, and contrast this with the effects of task repetition on behavioral measures of performance. More specifically, we used functional near infrared spectroscopy (fNIRS), which allows the estimation of relative changes in the concentration of oxygenated (oxy-Hb) and deoxygenated hemoglobin (deoxy-Hb) and electrodermal activity (EDA), a commonly used peripheral marker of cognitive and affective processes related to limbic and paralimbic activities. A multimodal approach with cortical (fNIRS detecting changes in hemodynamic activity of the prefrontal cortex), peripheral (EDA accessing the changes in sympathetic activity), and behavioural measurements can provide a broad picture of the cognitive processes of interest (Tanida et al. 2007; Hoshi et al. 2011; Doi et al. 2013; Tupak et al. 2014; Balconi et al. 2015a, b, Sklivanioti Greenfield et al. 2022, Sklivanioti Greenfield et al. 2023). We hypothesized that (i) task repetition would improve immediate and delayed performance and lead to habituation of sympathetic electrodermal activity and (ii) that escitalopram would preferentially improve performance, reduce prefrontal activity and sympathetic arousal during cognitive control in an emotional setting compared to cognitive control in non-emotional setting.

## Methods

### Participants

Study participants [*n* = 23 in the escitalopram arm (48% females, mean age 30.5 years, SD 7.7 years) and *n* = 18 in the placebo arm (50% females, mean age 31 years, SD 8.2 years)] were recruited from non-clinical population by advertisement in Psychiatry Southwest and Karolinska University Hospital, Huddinge, Sweden. The sample size was calculated after performing a power analysis based on a pilot fNIRS study and previous related fMRI studies, see also Supplementary material. Prior to the first trial, an overview of the general scope of the study and the outline of the experimental procedure were given to all participants until it was made sure that they fully understood the procedure. All participants met the following inclusion criteria: able and willing to provide written informed consent, > 18 years of age or older at the time of recruitment, free of any psychiatric, neurological and addiction disorders as well as any current drug use including psychoactive medication. All subjects were asked to abstain from alcohol consumption at least one day prior to the trial and were instructed to continue their usual consumption of coffee and nicotine and keep it the same level prior to each part of the testing.

### Ethics

The study was approved by the Stockholm County’s ethics committee (Dnr 2008/1397–31/4, 2013/722–32 and 2022–02605-02). All subjects were given verbal and written information and gave written informed consent through their signature prior to the start of the experiment, in accordance with the Declaration of Helsinki.

#### Study design

##### Experimental design

**Fig. 1:**
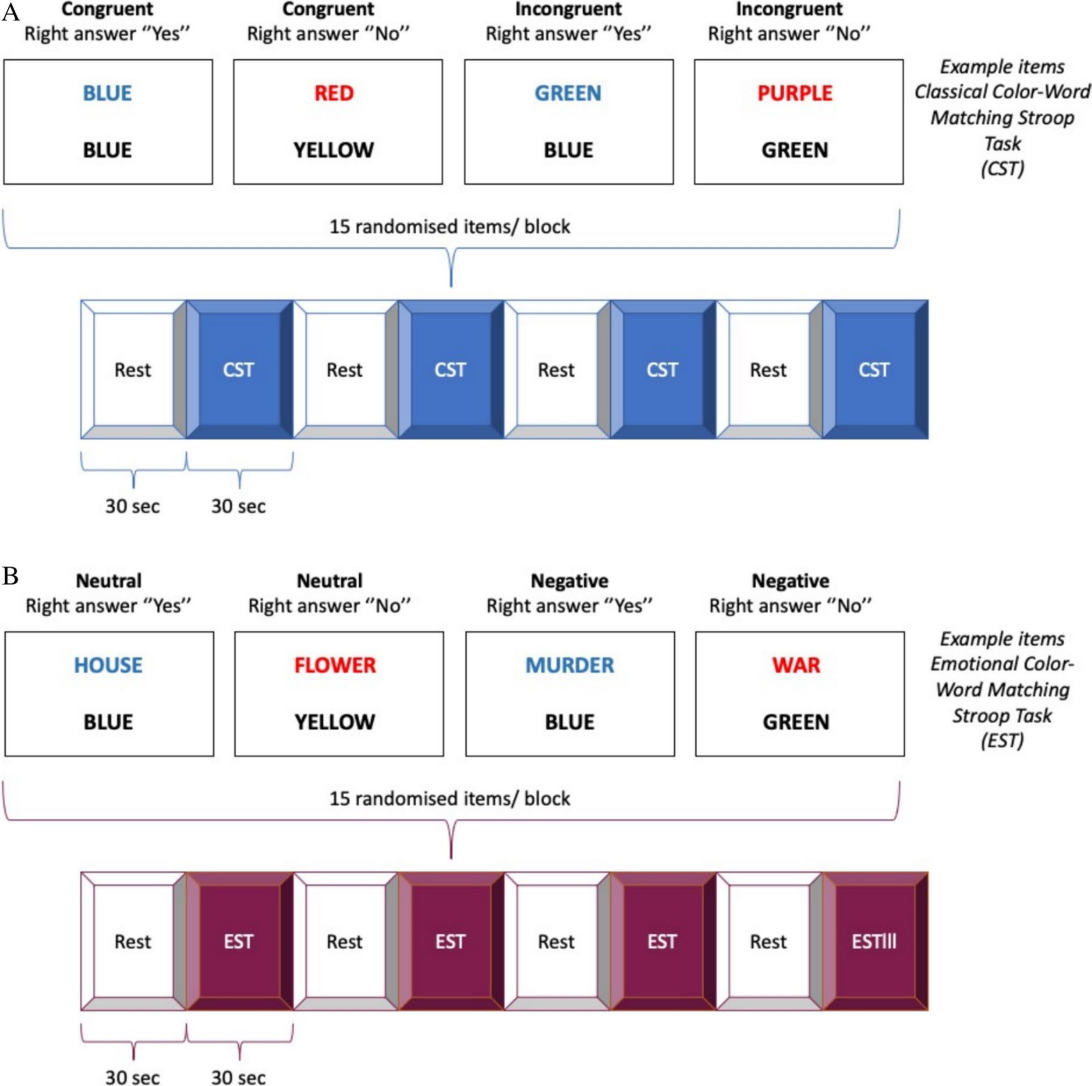

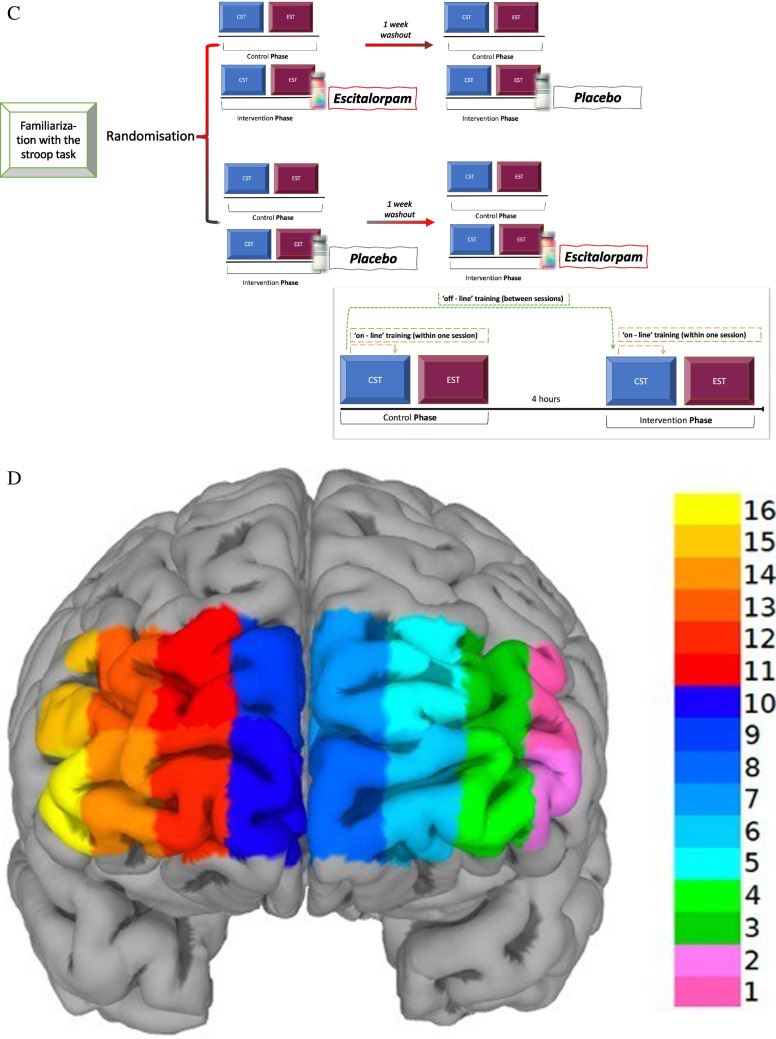
**A-D** Paradigm timing for each task, classical Stroop test (A) and emotional Stroop test (B). (C) Shows the whole experimental setting (D) Corresponding fNIRS channel locations on the PFC. The data were averaged over left (LPFC, Channels 1–6), medial (MPFC, Channels 7–10) and right (RPFC, Channels 11–16) prefrontal regions to increase signal-to-noise ratio

An adapted version of the Color–Word interference task (Treisman and Fearnley 1969) similar to the Color-Word Matching Stroop Task described by Zysset et al. (2001) was used. Participants were presented two rows of letters and were instructed to determine, by button-press, if the color of the top row letters corresponded to the color name written at the bottom row (Fig. 1A-B). The experimental setup included a block design of two tasks: *cognitive Stroop task (CST)* and *emotional Stroop task (EST)*. For the cognitive Stroop task interference was introduced when the color of the top word and the lexical information contained did not match (incongruent trials). For the emotional Stroop task interference was introduced by using emotionally charged words with negative valence (negative trials). The CST thus comprised of congruent and incongruent trials and the EST of trials with neutral words and negatively charged words. Each task included four blocks, each block was 30-s long and was preceded by a 30-s long period of *Rest*. In each block, 15 trials were presented, randomized between congruent and incongruent trials for CST and neutral and emotionally charged words for the EST (Fig. 1A-B). ‘On-line’ practice was defined as real-time change in performance or electrodermal activity during an ongoing task. Delayed or ‘off-line’ effect of task repetition was defined as the change in performance or electrodermal activity four hours after the first session. (Fig. 1C). Experiments were performed in a secluded room with dimmed lighting, stable temperature and humidity and measurement was started at least 15 min after participants had acclimatized to the room connected to the fNIRS device and feeling relaxed and comfortable. The tasks were implemented in E-Prime (version 2.1, http://www.pstnet.com/eprime.cfm).

##### Pharmacological intervention

The participants arrived in the morning, performed the experiment in the absence of pharmacological intervention (*Control*), and then ingested a single dose of 10 mg escitalopram or placebo in a randomized blinded fashion, see Fig. 1c. Approximately four hours later, participants returned to perform the same experimental procedure again in a second session (*Intervention*). Intervention consisting of 10 mg escitalopram was given in the form of Cipralex® oral drops mixed in a glass of water (350 ml). For placebo, participants received the same amount of water with no escitalopram added to it. No specific expectation relating to the possible effects of placebo was discussed with participants. To control for blinding, participants were asked to hazard a guess as to which of the interventions they thought they had received after the experiment was over. Participants were also asked to freely state any type of side effect they experienced.

#### Data acquisition and analysis

##### Behavioral measures

The behavioral data were recorded using E-prime software version 2.1 (Psychology software tools, Sharpsburg, PA, USA) and data on reaction time and correct answers were extracted were extracted. As previously mentioned, extensive research has established that successful performance on the Stroop task requires the engagement of cognitive control processes in the brain. In this study, we utilized both the Classical Cognitive Stroop Task (CST) and the Emotional Stroop Task (EST) to elicit and engage these processes. High accuracy scores ensured that cognitive control was successfully activated. In addition to error rates (a proxy for accuracy), reaction times were measured as an index of cognitive control efficiency, where"reaction"refers to a response requiring cognitive control rather than mere perceptual processing. Premature responses were defined as responses with reaction time < 200 ms. These trials were used to assess as surrogate measures of impulsivity in an exploratory manner but were otherwise excluded from further analysis.

##### Functional near-infrared spectroscopy (fNIRS) recordings

A continuous wave fNIRS device consisting of a flexible headband holding light sources and detectors in fixed distributions placed on the forehead of the participant and connected to a fNIR100 data acquisition box with sampling rate of 2 Hz was connected to a personal computer via an MP150 data acquisition and analysis system (Biopac Systems Inc, JOR AB Sweden) and used to measure the relative changes in the concentration of oxy-hemoglobin (Δoxy-Hb). The sensor consisted of four infrared light sources emitting at two different wavelengths (730 and 850 nm) and ten detectors separated by a distance of 2.5 cm, giving a total of 16 channels for recording different parts of the prefrontal cortical mantle (mainly bilateral BA 9, 10, 45, 46 (Ayaz et al. 2013), see Fig. 1D). Electrode placement was done according to the protocol recommended by Biopac Systems Inc and as described by Ayaz and colleagues (Ayaz et al. 2011) and visualized by Ayaz et al. (2013) in their Fig. 7. After lifting the hair off the forehead, the sensor strip was placed just above the eyebrows and the center of the sensor was matched with the vertical axis of symmetry that passes through the nose. Data acquisition was performed using the COBI Studio software (Cognitive Optical Brain Imaging Studio, fNIRS Devices LLC) and a second personal computer was connected to the system via a COM cable to synchronize the E-Prime data set with the fNIRS and EDA data sets using Acqknowledge software v4.2 (Biopac Systems Inc, JOR AB Sweden). Raw light intensity data was automatically converted to levels of oxygenated hemoglobin (oxy-Hb) by COBI software utilizing a modified Beer-Lambert Law.

##### Preprocessing and statistical analysis of fNIRS

NIRS-SPM toolbox (Tak et al. 2016) that utilizes the SPM12 package (Wellcome Department of Cognitive Neurology, London, UK) and runs under MATLAB (MATLAB_R2019b, Mathworks, Natick, MA) was used to analyze the fNIRS data. Oxy-Hb was analyzed, as it measures more reliably cerebral blood flow and task-related activation (Hock et al. 1995; Tanida et al. 2004; Kameyama et al. 2006; Tanida et al. 2007; Bendall et al. 2016), whereas deoxy-Hb is known to suffer from low signal–noise ratio limiting its usability (Balconi et al. 2015a, b; Tam and Zouridakis 2015; Bulgarelli et al. 2018). Physiological noise from respiration and cardiac pulsation was removed using two band-stop filters (0.12–0.25 and 0.7–2.0 Hz). Visual inspection of data showed minimal motion artifacts and comparison of data with and without motion correction (Scholkmann et al. 2010) showed no substantial differences, therefore no correction for movement artefacts was performed. Less than 5% of the trials in all channels (without specific channels being overrepresented) in all trials were excluded because of technical quality problems. For detrending and reducing low-frequency confounders, a high-pass filter based on a discrete cosine transform set with the cut-off period set to 128 s was utilized. Autocorrelations in the time series due to hemoglobin changes were corrected using pre-whitening that is available in the NIRS-SPM toolbox (Purdon and Weisskoff 1998). Generalized linear model (GLM) was used to separately fit data from each channel to ideal responses modelled through the onset timings, convolved with the hemodynamic response function (HRF) consisting of the canonical HRF and its temporal and dispersion derivatives. T-contrasts for task vs rest were calculated for each channel and each task: 1) cognitive Stroop blocks (CST) vs. rest [CST – REST] and 2) emotional Stroop blocks (EST) vs. rest [EST – REST]. As a result, channel-specific beta coefficients were generated which were used for further statistical analyses. Data were averaged over left (channels 1–6), medial (channels 7–10) and right PFC (channels 11–16) to improve signal–noise ratio, Fig. 1D. A post-hoc channel-wise analysis of the fNIRS data was performed where nominal testing was applied (Moyé 2008). Outlier correction was performed by replacing outliers with the Q1 − 1.5 IQR and Q3 + 1.5 IQR rather than outright removing them, as a more conservative approach.

##### Electrodermal activity (EDA)

Two non-polarizable Ag–AgCl electrodes (EL 507, JOR AB Sweden) were placed on the middle phalanges of digits 2 and 3 of the left hand to record electrodermal activity using GSR100C amplifier, Biopac MP150 system, and captured with Acqknowledge software v4.2 (Biopac Systems Inc, JOR AB Sweden). Amplifier gain was set at 10 μmho/V, low-pass filter at 1 Hz and high-pass filter at 0.05 Hz. To remove artefacts and high-frequency noise, a low-pass filter (5th-order low-pass Butterworth filter with cut-off frequency at 1 Hz) and a median smoothing (smoothing window equal to the sample frequency 8 Hz) were applied to the raw EDA signals after acquisition. Subsequently the pre-processed signal was decomposed into three components: tonic EDA signal, phasic EDA signal and white Gaussian noise using a convex optimization approach (Greco et al. 2016). Lastly, using MATLAB’s EDA Toolbox (available at https://github.com/mateusjoffily/EDA/wiki), frequency of phasic electrodermal activity (stimulus-induced electrodermal response, here abbreviated as EDR) was calculated for the eight rest periods (non-specific EDR), and for the stimulus-evoked cognitive and emotional Stroop task periods. Block EDR frequency was used for the secondary analysis related to task repetition/practice sessions. Latency window for the stimulus-evoked EDR between 1–3 s after stimulus onset.

##### Statistical analyses

Outcome measures were a priori defined for the behavioral (reaction time), fNIRS (region-wise analysis of left, right and medial prefrontal cortex) and EDA (frequency of phasic electrodermal response (EDR)) experiments. A block-design analysis was used to assess differences between phases and conditions with a set of multilevel, mixed-effect, linear regression models (phase (control vs intervention), drug (placebo vs escitalopram), time (continuous); and random effects: intercepts for participants due to repeated measures). Maximum likelihood estimation was applied to the dependent primary outcomes (behavioral, fNIRS and EDA) and the Benjamini–Hochberg method used to correct for multiple comparison (significant raw *p*-values are reported). All statistical analysis was performed using Stata 14 software (StataCorp. 2015. Stata Statistical Software: Release 14. College Station, TX: StataCorp LP). See Supplementary material, for detailed report of the statistical analyses and results.

## Results

### Behavioral data

#### Delayed effects of practice and effect of escitalopram on this

**Fig. 2:**
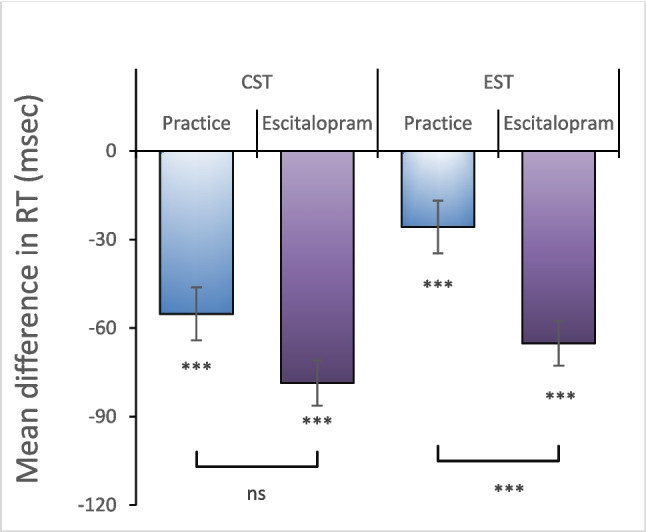
Practice led to significant delayed or “off-line” improvement in performance, as measured by reduction in mean reaction time during the second session four hours later (placebo). In cognitive Stroop, escitalopram had no additional effects on this, while during emotional Stroop, escitalopram significantly improved performance compared to placebo. CST; cognitive Stroop, EST: emotional Stroop

To investigate delayed effects of task-repetition four hours after the first session, accuracy and reaction time were analyzed with a three-factor (phase, drug, phase x drug) linear mixed model. Error events were rare and accuracy high, and we found no significant effects in these in all analyses performed. For reaction time, there was a main effect for phase (*p* < 0.001) during the cognitive Stroop task, but not for phase x drug interaction (*p* = 0.054), indicating that there was significant improvement in performance four hours after the first session, which was not however significantly modified by escitalopram. For emotional Stroop on the other hand, there was a main effect for phase (*p* < 0.001) and for phase x drug interaction (*p* < 0.001), such that escitalopram, in addition to the delayed performance improvement caused by practice, further improved performance compared to placebo (Fig. 2).

#### ‘On-line’ improvement and effects of escitalopram and placebo on this

**Fig. 3:**
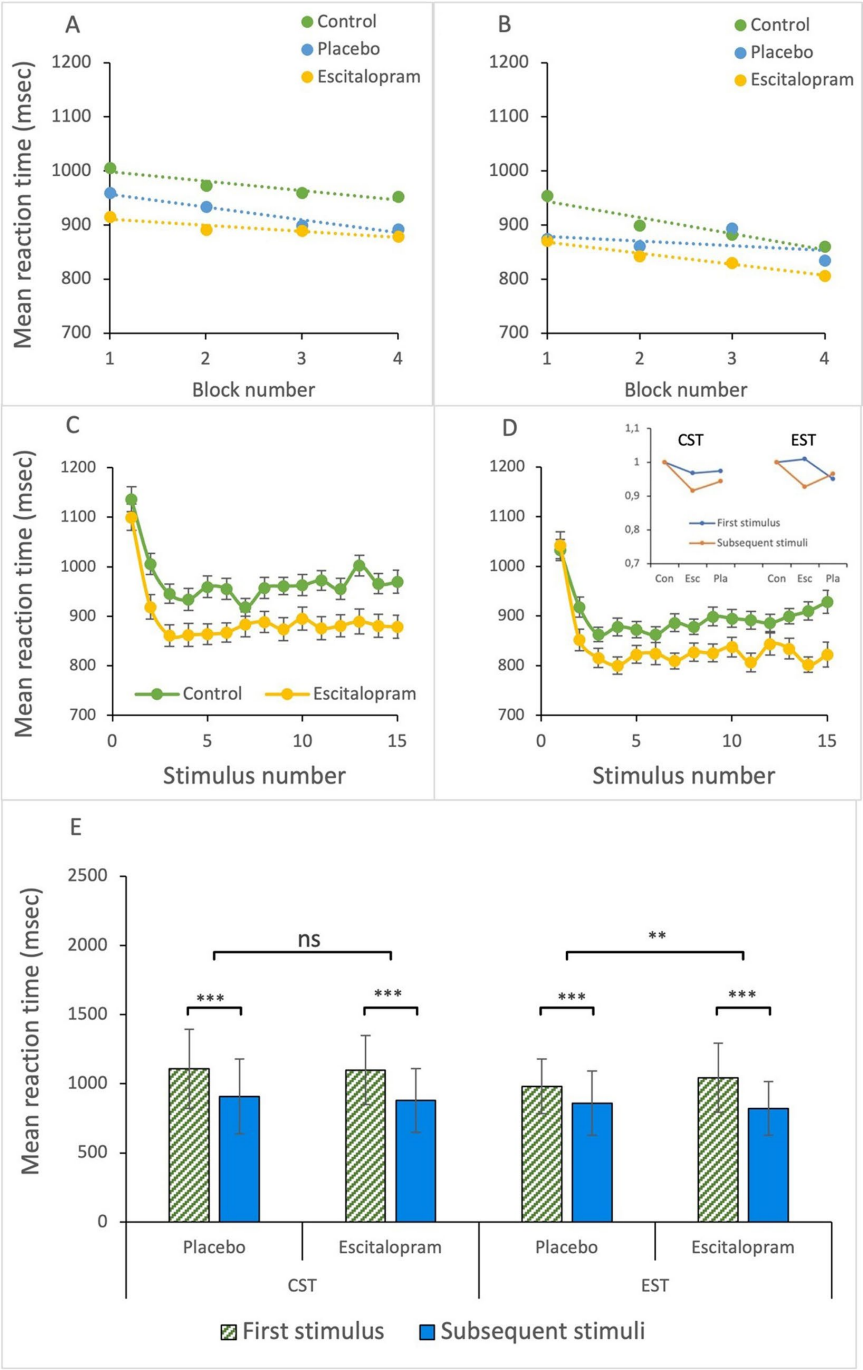
**A-B** Continuous decrease in reaction time with increasing block number. Shown is mean reaction time for each block (average of 15 stimuli) during cognitive (A) and emotional Stroop (B) in control or after ingestion of escitalopram or placebo, as indicated. **C-D** Reaction time for individual stimuli averaged across the four blocks for cognitive (C) and emotional Stroop (D). Inset in 3D shows relative effects of escitalopram (Esc) and placebo (Pla) on the first stimulus and subsequent 14 stimuli, during cognitive (CST) and emotional Stroop (EST) tasks compared to control (Con). **E** Effect of escitalopram and placebo on the first stimuli and subsequent 14 stimuli. Escitalopram had no significant effect on the first stimulus but compared to placebo significantly decreased reaction time starting from the second stimulus during emotional but not cognitive Stroop

To study ‘on-line’ effects of task repetition within an ongoing task, reaction time was analyzed with a three-factor (phase x drug x time) linear mixed model. We found significant main effect for time (*p* < 0.001) in both the cognitive and the emotional Stroop tasks, indicating a significant reduction in reaction time with increasing number of stimulus both within and between blocks. The rate of the block-by-block reduction in reaction time was not significantly modified by escitalopram compared to placebo in either cognitive (*p* = 0.226) or emotional (*p* = 0.207) Stroop tasks (Fig. 3A-B). When we looked pulse by pulse at the individual stimuli within a block with a three-factor (drug x phase x order) linear mixed model, reaction time for the first stimulus was significantly longer compared to that of subsequent stimuli in both cognitive and emotional Stroop tasks (*p* < 0.001, Fig. 3C-D). Escitalopram, compared to placebo, improved significantly more the reaction time for the stimuli 2–15, compared to the first stimulus in the block, during emotional (*p* < 0.001) but not cognitive Stroop task (*p* = 0.068, Fig. 3E).

#### Effects of escitalopram and placebo on premature responses

**Fig. 4:**
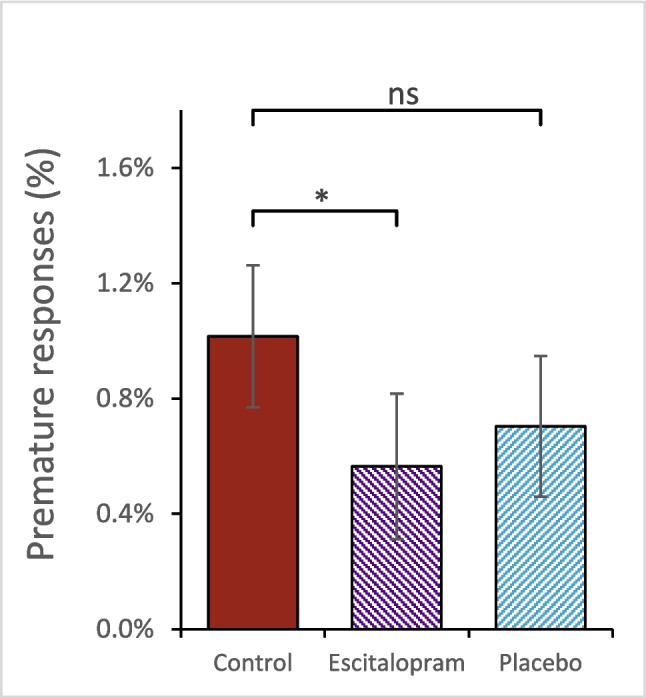
Premature responses (defined as reaction time of 200 ms or less). Escitalopram, but not placebo, significantly decreased these compared to control

Exploratory analysis was performed on impulsive behavior using premature responses as surrogate marker. Mixed-effects logistic regression was applied to assess if escitalopram had significant effects on premature responses, defined as reaction time of 200 ms or less. Premature responses were aggregated across both cognitive and emotional Stroop tasks and amounted to roughly 1% of total responses. Escitalopram significantly reduced this compared to control (OR 0.57, CI 95% 0.33–0.95, *p* = 0.032), while placebo had no such effect (OR 0.74, CI 95% 0.41–1.32, *p* = 0.306, Fig. 4).

### Functional near-infrared spectroscopy recordings (fNIRS)

**Fig. 5:**
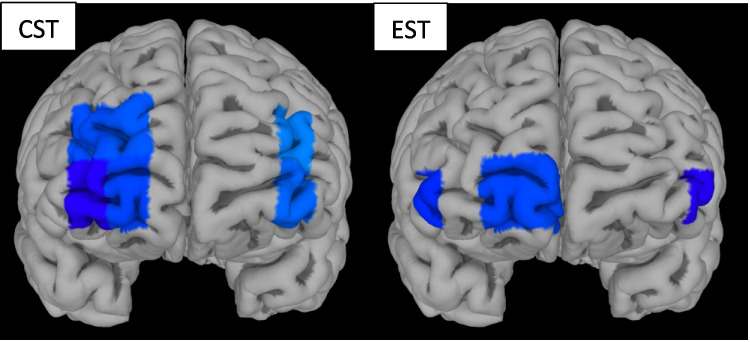
**A**-**B** Differences in Δoxy-Hb levels between control and escitalopram trials. In both tasks, a reduction in activation was recorded, in dorsolateral and ventrolateral channels for cognitive Stroop and in ventral and medial channels for emotional Stroop. There were no significant effects of placebo under any condition. CST: Cognitive Stroop task, EST: Emotional Stroop task

During cognitive Stroop, there was significant reduction in activation in right prefrontal cortex (PFC) after ingestion of escitalopram compared to control (*p* = 0.022). Post-hoc channel-wise analysis showed significant deactivation in dorsolateral and ventrolateral PFC channels (CH 3, 4, 11, 12, 13, 14; Fig. 5A), but not medial channels. In the emotional Stroop task, there was even more wide-spread reduction in PFC activation after escitalopram in right (*p* = 0.031), medial (*p* = 0.043) and left PFC (*p* = 0.013). Secondary post-hoc channel-wise analysis showed deactivations in ventrolateral and ventromedial channels after escitalopram (CH 2, 10, 12, 16, Fig. 5B), but not dorsal channels. Placebo had no significant effects on PFC activity, neither during cognitive nor during emotional Stroop tasks.

### Electrodermal activity (EDA)

#### Effects of escitalopram and placebo on mean EDR frequency

To evaluate the overall effects of escitalopram and placebo on electrodermal activity, mean frequency of stimulus-evoked phasic electrodermal response (EDR) was averaged block-wise or for the whole task (four blocks). No significant change in EDR frequency was found for any task under any condition compared to control.

#### Within session effects of practice (task repetition)

**Fig. 6:**
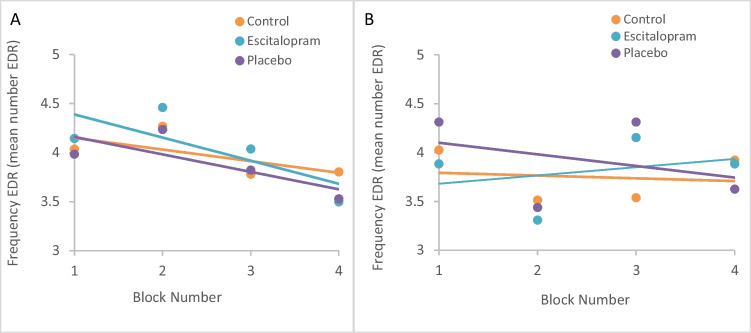
**A**-**B** Electrodermal activity recordings, effects of task repetition within an ongoing task. Frequency of phasic electrodermal response (EDR) was significantly reduced with task repetition during cognitive but not emotional Stroop task reflecting habituation. Shown is mean EDR frequency per block and linear best fit over time

To evaluate the effects of on-line task repetition, stimulus-evoked EDR were evaluated on an block-by-block basis. In cognitive Stroop, EDR frequency decreased significantly with increasing block number under all conditions tested (control *p* = 0.022, escitalopram *p* = 0.001, placebo *p* = 0.031), without any significant difference between the conditions. There was no such decline in EDR frequency during emotional Stroop, neither in control phase (*p* = 0.758) nor during the two intervention phases (*p* = 0.450 & *p* = 0.474 for escitalopram and placebo, respectively), Fig. 6A-B.

## Discussion

Cognitive control is crucial for goal-directed behaviour and for emotional well-being. Enhancing elements of cognitive control through behavioral intervention, such as physical exercise (Neudecker et al. 2019; Sun et al. 2022), mindfulness (Li et al. 2018), strategy training (Janowich and Cavanagh 2018) of pharmacological interventions (Sahakian and Morein-Zamir 2007; Husain and Mehta 2011; Kapur 2020; Roberts et al. 2020b, a) has been widely studied in clinical and non-clinical settings (Linssen et al. 2014; Ilieva et al. 2015; Marraccini et al. 2016). Acute and delayed effects of task repetition (Stagg 2021), and whether practice in a specific cognitive domain confers improvement in other domains has also been studied (Davidson et al. 2003; Schweizer et al. 2011). However, which specific aspects of cognitive control are amenable to which specific cognitive enhancing strategies and under what conditions is not fully understood. Regarding pharmacological intervention, for example, the role of serotonergic intervention in cognitive control is not as well studied as that of catecholaminergic intervention. The aim of the present study was to investigate how single-session task repetition and single-dose serotonergic intervention with escitalopram would modulate cognitive control in emotional and non-emotional settings.

### Effects of practice with task repetition

Immediate and delayed effects of task repetition were investigated during “hot” and “cold” cognitive control conditions using emotional and cognitive Stroop tasks, respectively. We hypothesized that task repetition would improve performance in both tasks but would cause greater habituation of arousal/electrodermal activity during emotional Stroop compared to cognitive Stroop. Indeed significant ‘online’ and ‘off-line’ improvement in performance, as measured by reduction in reaction time, were observed for both tasks. But contrary to our expectation, the frequency of stimulus-evoked phasic electrodermal responses (EDR) habituated significantly during cognitive but emotional Stroop task. A plausible explanation for this might be that learning to ignore threatening cues might not be advantageous for survival and, as such, less amenable to modulation by habituation, as emotion induction with threatening stimuli has been shown to be prioritized and command attention at the expense of the color naming task (Ohman et al. 2001; Sharma and McKenna 2001; McKenna and Sharma 2004). Evidence from various tasks also suggests that emotional information is highly salient and does not easily fade away with habituation (Gray 2004; Schweizer et al. 2011), in a way that reminds of the distally analogous rapidly (light tough) and slowly (pain) adapting somatosensory systems (Koerber and Mendell 1988; Birder and Perl 1994).

### Effects of escitalopram

Salient events shape PFC activity by inducing release of neuromodulators that coordinate and fine-tune cognitive processes to match environmental demands (Robbins 2005). Modulation of catecholaminergic and cholinergic transmission markedly affects dorsolateral PFC function, whereas serotonergic modulation is assumed to heavily modulate ventral and medial PFC (Cools and Arnsten 2022). Dorsolateral PFC is involved in biasing attention towards task relevant information and suppression of task-irrelevant distractions (Chan et al. 2008, Fitzgerald et al. 2018), and together with dorsal ACC (anterior cingulate cortex) in resolving cognitive conflicts (Banich et al. 2009) and, as such, is regularly activated during Stroop tasks (Anderson et al. 2008; Pessoa 2008; Banich et al. 2009). On the other hand, ventromedial PFC is thought to have greater role in motivation and emotion processing (Damasio 1996; Phan et al. 2002) and in top-down regulation of negative emotions (Phan et al. 2004), and is activated by emotion—cognition interactions (Sebastian et al. 2017). Serotonergic modulation is implicated in many processes of importance for cognitive control, including constraining impulsive choice and enabling waiting (Robbins and Crockett 2010; Ye et al. 2014; Skandali et al. 2018), regulating response to aversive stimuli (Robbins and Crockett 2010), reducing hypervigilance (Ramaekers et al. 1995, O'Hanlon et al. 1998; Schmitt et al. 2002) and enhancing reversal learning (Clarke et al. 2004; Clarke et al. 2005; Walker et al. 2008; Brown et al. 2012; Rygula et al. 2014; Kanen et al. 2019; Roberts et al. 2020b, a). It has also been speculated that SSRI might act by filtering aversive emotions from reaching conscious awareness (Del-Ben et al. 2005) and this way reduce the cost of cognitive processing of negative emotions (Murphy et al. 2009; Lochner et al. 2012; Wolf et al. 2018). Based on these assumptions, we hypothesized that acute administration of escitalopram would have greater effect in modulating functions related to ventral and medial PFC than functions related to dorsolateral PFC. More specifically, we hypothesized that escitalopram would affect emotional Stroop to a greater extent than cognitive Stroop, both behaviorally and in terms of PFC activations. Consistent with this, we observed greater effect of escitalopram during emotional Stroop both in terms of behavioral and cortical measures of cognitive control. There was more widespread deactivation during emotional Stroop (right, medial and left PFC) compared to cognitive Stroop (right PFC). SSRI even after single dose are known to reduce emotional response to salient stimuli (Kent et al. 1998, Sklivanioti Greenfield et al. 2023), which could be one way how serotonergic manipulation improved performance in the emotional Stroop task. Broad deactivations with a ventral and medial PFC dominance were found during emotional Stroop after escitalopram, reflecting that the emotional distraction might have been ameliorated by escitalopram consistent with the improved task performance at the behavioral level. For cognitive Stroop, deactivation in right PFC mainly covered dorsolateral PFC and inferior frontal gyrus, areas which are often activated during Stroop task (Levy and Wagner 2011) and are known to be involved in inhibitory control (Aron et al. 2004; Sundermann and Pfleiderer 2012).

Although the dynamics of serotonin firing during this Stroop task in humans remain unclear, a speculative interpretation could be proposed as follows: For escitalopram to effectively inhibit serotonin reuptake, there must be ongoing serotonin release, otherwise there will be no uptake to inhibit. Given that the dynamics of serotoninergic neuronal firing in this context is not yet fully understood, we tentatively propose a qualitative difference between the first stimulus of each block and subsequent stimuli. At the start of the task, it can be hypothesized that only baseline tonic serotonergic activity is present, providing the setting for the first stimulus. After this initial stimulus, a process likely begins—conceivably involving phasic serotonergic activity—which could serve as the substrate for manipulation by SSRIs. While the precise temporal dynamic of this process is not known, we assume that serotonergic activity remains relatively stable over the course of a block. Indeed, the fact that escitalopram had no effect on the first stimulus, but significantly reduced reaction time of subsequent stimuli seems to be consistent with this. Serotonin activity at the synapse is terminated by reuptake and because of interplay with presynaptic feedback regulation, uptake inhibitors will affect extra- and intrasynaptic serotonin levels differently, just as is the case at noradrenergic and dopaminergic terminals, where uptake blockers increase extra- but not intrasynaptic transmitter levels (Msghina et al. 1992; Suaud-Chagny et al. 1995). If the effect of serotonin on the emotional Stroop task was mediated non-specifically by extra-synaptic receptors, one would expect even the first stimulus to be affected, since there will be non-specific serotonin release here and there that would increase extra-synaptic serotonin levels in the presence of escitalopram. If, however, the effect of serotonin on the emotional Stroop task is mediated specifically by stimulus-evoked serotonin release at intrasynaptic receptors, then serotonin would have no effect on the first stimulus but would modulate the emotional Stroop task starting from subsequent stimuli, which was the case in the present study. In the cognitive Stroop task, we found no overall effect of escitalopram on performance at the behavioral level, although we found significant reduction of prefrontal activity at the cortical level. The discrepancy here is compatible with the idea that the wide-spread cortical effects of escitalopram seen during both cognitive and emotional Stroop tasks might be mediated by more general, stimulus non-specific increase of extra-synaptic serotonin levels.

As mentioned above, serotonin modulates impulsive choice, enhancing the ability to wait rather than responding prematurely (Robbins and Crockett 2010; Ye et al. 2014; Skandali et al. 2018). Consistent with this, we found that escitalopram, but not placebo, significantly reduced premature responses. In our study, premature responses were rare so that we had to aggregate these under both cognitive and emotional Stroop tasks to gain statistical power. Notwithstanding this, our results, although exploratory in nature, indicate that increasing serotonergic transmission with escitalopram leads to enhanced ability to withhold impulsive responses and wait for the appearance of stimulus before prematurely reacting.

In summary, our study suggests that enhancing serotonergic transmission with single dose escitalopram enhances cognitive control when interference has emotional content by filtering the emotional contents in a phasic manner and not by generally and tonically modifying cognitive control and associated peripheral arousal.

### Strengths and limitations

The study employs a multimodal approach including behavioral, cortical, and peripheral measures that investigate cognitive control from different angles. Furthermore, we employed and contrasted two well-validated tasks that are similar in structure but differ in type of interference employed, a cognitive vs. an emotional interference during an ongoing cognitive task. A major limitation in extrapolating these results to reach clinical conclusions is that acute administration of escitalopram was used and it should be remembered that single-dose SSRI can yield different, even opposite results compared to repeated treatment with the same medication (Anderson et al. 2008). Furthermore, the fact that fNIRS does not capture changes in subcortical activations because of low cortical penetration also limits the interpretation of the results. Lastly, to study the effect of task repetition, we had only two arms after the first session, escitalopram, and placebo. Although we tried to minimize expectation in the placebo arm, we feel we would have needed a third arm when the task was repeated with participants having to ingest neither placebo nor escitalopram, so that we could study the bare off-line effects of task repetition.

## Conclusions and implications

Our aim was to investigate the acute effects of serotonergic manipulation on emotional and non-emotional aspects of cognitive control, and a indeed a differential effect was found. Task repetition improved performance in both cognitive and emotional Stroop tasks, while arousal, as measured by electrodermal activity, habituated only when the interference was caused by cognitive conflict and not by emotionally salient negative stimuli. On the other hand, acute administration of single dose SSRI was associated with gains in performance only when the interference had emotional content, but only starting from subsequent stimuli and not on the first stimulus, indicating that intrasynaptic rather than extra-synaptic serotonin levels and serotonin receptors might have been at play here.

## Supplementary Information

Below is the link to the electronic supplementary material.

Supplementary file1 (PDF 301 KB)

## Data Availability

The raw data supporting the conclusions of this article will be made available by the authors on request, without undue reservation.
