# Supplementary material for: Behavioral intervention with task repetition compared to pharmacological intervention with SSRI for enhancement of cognitive control in emotional and non-emotional settings
Source: Psychopharmacology (Berl). 2025 Jun 19;242(12):2817–33. doi: 10.1007/s00213-025-06835-6 (PMC12675703; doi:10.1007/s00213-025-06835-6)
Supplement: Supplementary file 1 — Supplementary file1 (PDF 301 KB) [file 213_2025_6835_MOESM1_ESM.pdf]

Supplementary Material

Behavioral intervention with task repetition compared to pharmacological intervention with SSRI for enhancement of cognitive control in emotional and non-emotional settings – Sklivanioti, Wang, Msghina

1 Study plan

Primary analyses	Variables	Research questions	Model	Operationalization
'off-line' effect	**Primary:**<br>Mean RT<br>fNIRS (LPFC. MPFC. RPFC)<br>Frequency EDR<br>**Secondary:**<br>fNIRS (16 channels)	Does practice (placebo) have a significant effect 4hours later when the tasks is repeated?	Drug##Phase (mixed-effect linear regression model)	Phase main effect
		Does escitalopram and placebo have significantly different effects?		Drug##Phase interaction effect
'on-line' effect	RT (event related analysis)	Does task repetition improve performance on-line?		Time main effect

Behavioral intervention with task repetition compared to pharmacological intervention with SSRI for enhancement of cognitive control in emotional and non-emotional settings – Sklivanioti, Wang, Msghina

		Does escitalopram and placebo have significantly different effects on the slope of the improvement?	Time##Phase##Drug (mixed-effect linear regression model)	Drug(main effect) + Drug#Time(interaction effect) + Phase#Drug(interaction effect) + Phase#Drug#Time(interaction effect)
	Frequency EDR (block related analysis)	Does task repetition improve performance on-line?	Block##Phase##Drug (mixed-effect linear regression model)	Block main effect
		Does escitalopram and placebo have significantly different effects on the slope of the improvement?		Drug(main effect) + Drug#Block(interaction effect) + Phase#Drug(interaction effect) + Phase#Drug#Block(interactio n effect)
Effects of Order of the stimulus	RT (event related analysis)	Is there a significant differnce between the RT between the first and the subsequent items?	Order##Phase##Drug (mixed-effect linear regression model)	Order main effect
		Do escitalopram and placebo have differential effect on the first vs subsequent items?		Drug#Phase (main effect) + Order#Drug#Phase (interaction effect)

Supplementary Material

Behavioral intervention with task repetition compared to pharmacological intervention with SSRI for enhancement of cognitive control in emotional and non-emotional settings – Sklivanioti, Wang, Msghina

Secondary analyses

Premature responses	RT < 200msec	Does practice reduce the incidence of premature responses?	mixed-effects logistic regression	Phase main effect for escitalopram and placebo
		Does escitalopram reduce the incidence of premature responses?		
Comparison between tasks	Mean RT	Mean RT in CST compared to EST in control conditions	Stroop (interaction effect)##Phase##Drug (mixed-effect linear regression model)	Stroop main effect
		Mean RT in CST compared to EST when placebo was given		Phase#Stroop interaction effect
		Mean RT in CST compared to EST when escitalopram was given		Phase#Stroop (interaction effect) + Phase#Drug#Stroop (interaction effect)

Phase	control vs intervention
Drug	placebo vs escitalopram
Time	continuous
Order	first vs not-first (subsequent) item

Behavioral intervention with task repetition compared to pharmacological intervention with SSRI for enhancement of cognitive control in emotional and non-emotional settings – Sklivanioti, Wang, Msghina

main effects and interaction effects
RT: reaction time. only RT> 200 were analysed
LPFC: left prefrontal cortex
RPFC: right PFC
MPFC: medial PFC
EDR: stimulus-induced phasic electrodermal activity

---

2 Results

2.1 Behavioural data

Cognitive Stroop task	Dependent variable	Effects of interest (linear mixed models)	Coef	SE	z	P>|z|	95% CI (LL)	95% CI (UL)
	Mean RT	Phase main effect	-4.075	45.685	-0.090	0.929	-93.617	85.467

Behavioral intervention with task repetition compared to pharmacological intervention with SSRI for enhancement of cognitive control in emotional and non-emotional settings – Sklivanioti, Wang, Msghina

'off-line' effect								
		Drug##Phase interaction effect	-23.965	12.416	-1.930	0.054	-48.300	0.370
'on-line' effect	RT (event related analysis)	Time main effect	-0.813	0.367	-2.220	0.027	-1.532	-0.094
		Drug(main effect) + Drug#Time(interaction	56.038	48.188	1.160	0.245	-38.409	150.485

Behavioral intervention with task repetition compared to pharmacological intervention with SSRI for enhancement of cognitive control in emotional and non-emotional settings – Sklivanioti, Wang, Msghina

		effect) + Phase#Drug(interaction effect) + Phase#Drug#Time(interaction effect)						
Effects of Order of the stimulus	RT (event related analysis)	Order main effect	-150.072	26.866	-5.590	<0	-202.729	-97.415
		Drug#Phase (main effect) + Order#Drug#Phase (interaction effect)	-22.859	12.538	-1.820	0.068	-47.432	1.715
**Emotional Stroop task**	**Dependent variable**	**Effects of interest (linear mixed models)**	**Coef**	**SE**	**z**	**P>|z|**	**95% CI (LL)**	**95% CI (UL)**
‘off-line’ effect	Mean RT	Phase main effect	-25.913	8.163	-3.170	0.002	-41.913	-9.913

		Drug##Phase interaction effect	-39.117	10.736	-3.640	<0.001	-60.159	-18.075
'on-line' effect	RT (event related analysis)	Time main effect	-1.758	0.311	-5.650	<0.001	-2.367	-1.149
		Drug(main effect) + Drug#Time(interaction effect) + Phase#Drug(interaction effect) + Phase#Drug#Time(interaction effect)	-64.491	53.072	-1.220	0.224	-168.511	39.529
Effects of Order of the stimulus	RT (event related analysis)	Order main effect	-156.660	24.139	-6.490	<0.001	-203.971	-109.349
		Drug#Phase (main effect) + Order#Drug#Phase (interaction effect)	-44.876	10.811	-4.150	<0.001	-66.066	-23.686

	**Dependent variable**	**Effects of interest (mixed-effects logistic regression)**	**Coef**	**SE**	**z**	**P>|z|**	**95% CI (LL)**	**95% CI (UL)**
Premature responses	RT < 200msec	Phase main effect for escitalopram	0.696	0.207	-1.220	0.223	0.389	1.247

Behavioral intervention with task repetition compared to pharmacological intervention with SSRI for enhancement of cognitive control in emotional and non-emotional settings – Sklivanioti, Wang, Msghina

		Phase main effect for placebo	0.533	0.141	-2.380	0.017	0.317	0.895
	**Dependent variable**	**Effects of interest (linear mixed models)**	**Coef**	**SE**	**z**	**P>|z|**	**95% CI (LL)**	**95% CI (UL)**
Comparison between tasks	Mean RT	Stroop main effect	-84.025	8.963	-9.370	<0.001	-101.593	-66.457
		Phase#Stroop interaction effect	29.438	12.666	2.320	0.020	4.614	54.263
		Phase#Stroop (interaction effect) + Phase#Drug#Stroop (interaction effect)	13.480	10.822	1.250	0.213	-7.731	34.692

Supplementary Material

Behavioral intervention with task repetition compared to pharmacological intervention with SSRI for enhancement of cognitive control in emotional and non-emotional settings – Sklivanioti, Wang, Msghina

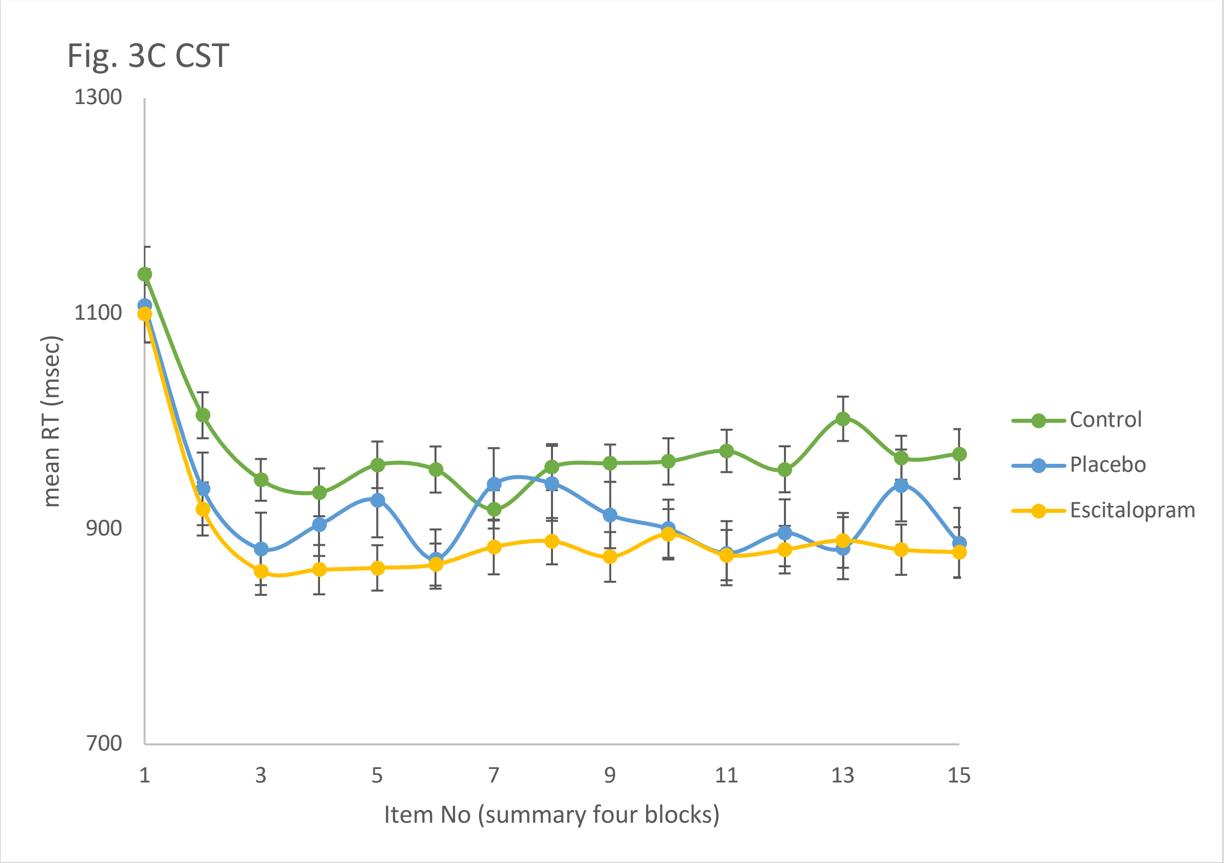

2.1.1 Fig. 3C and 3D

Behavioral intervention with task repetition compared to pharmacological intervention with SSRI for enhancement of cognitive control in emotional and non-emotional settings – Sklivanioti, Wang, Msghina

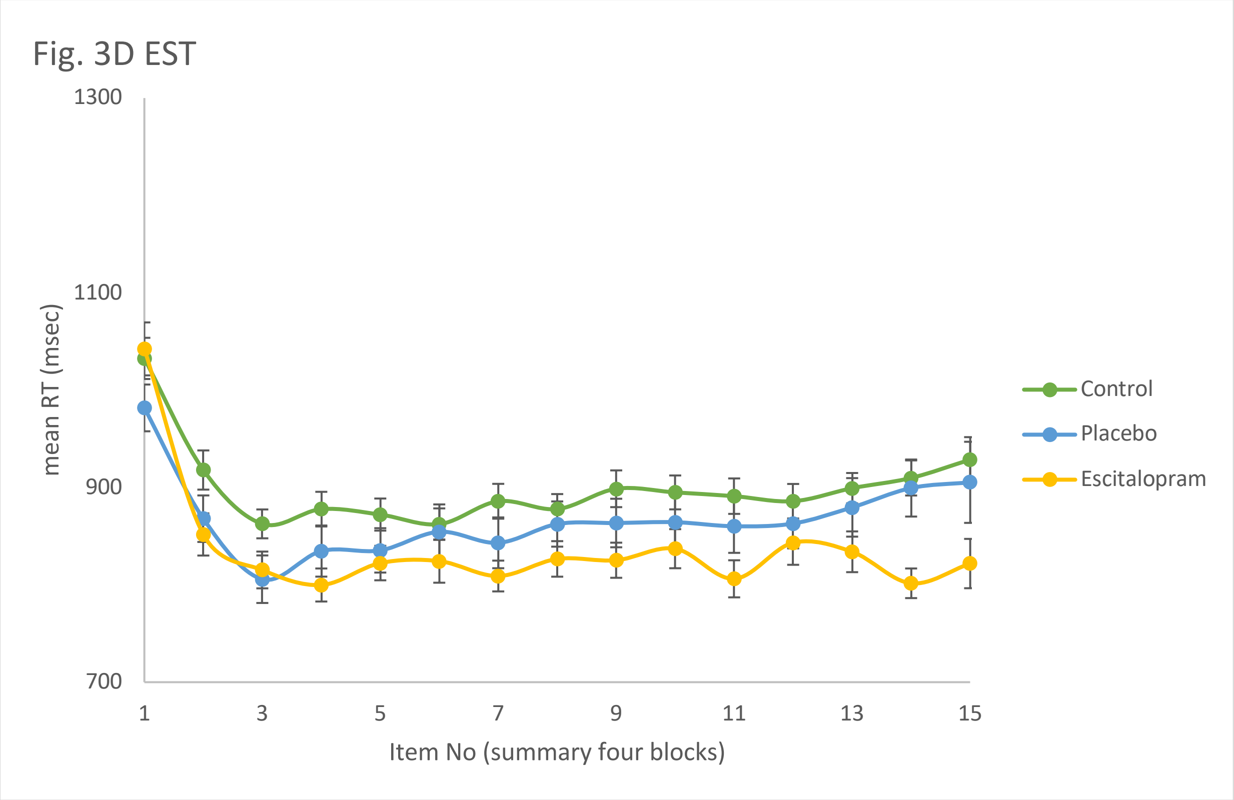

*(**3C-D**) Reaction time for individual stimuli averaged across the four blocks for cognitive (**3C**) and emotional Stroop (**3D**), figure including Placebo that was removed from the manuscript for visual clarity.*

2.2 fNIRS

Supplementary Material

Behavioral intervention with task repetition compared to pharmacological intervention with SSRI for enhancement of cognitive control in emotional and non-emotional settings – Sklivanioti, Wang, Msghina

2.2.1 Variables

Channels 1-6	Left prefrontal cortex (LPFC)	Primary outcome
Channels 7-10	Medial prefrontal cortex (MPFC)	Primary outcome
Channels 11-16	Right prefrontal cortex (RPFC)	Primary outcome
Channels 1-16	Individual channels	Secondary outcome

2.2.2 Results

2.2.2.1 Region-wise

fNIRS LPFC

Table (Linear Mixed Models)

Fixed effects	
Stroop	CST [0] and EST [1]
Phase	Control [0] and Pharmacological Intervention [1]
Drug	Placebo [0] and Escitalopram [1]
Random effects	Subject effect. due to repeated measures

Supplementary Material

Behavioral intervention with task repetition compared to pharmacological intervention with SSRI for enhancement of cognitive control in emotional and non-emotional settings – Sklivanioti, Wang, Msghina

Main effects and Interactions	Estimate	SE	z	P>|z|	95% CI (LL)	95% CI (UL)
STROOP	0.063	0.051	1.23	0.219	-0.038	0.164
PHASE	0.029	0.051	0.57	0.570	-0.071	0.129
STROOP#PHASE	0.025	0.073	0.34	0.735	-0.118	0.167
DRUG	0.105	0.062	1.68	0.093	-0.017	0.227
STROOP#DRUG	-0.006	0.067	-0.09	0.927	-0.138	0.126
PHASE#DRUG	-0.103	0.066	-1.56	0.120	-0.234	0.027
STROOP#PHASE#DRUG	-0.056	0.094	-0.59	0.554	-0.241	0.129
Intercept	-0.057	0.047	-1.21	0.225	-0.149	0.035
Wald chi2(7) = 17.21						
Prob > chi2 = 0.016						
ICC =0.45 (0.09 SE)						
**Contrasts of interest**						
**Effects of pharmacological intervention**						
**CST**						
Placebo contra Control	0.029	0.051	0.57	0.570	-0.071	0.129
Escitalopram contra Control	-0.074	0.043	-1.74	0.082	-0.158	0.009
**EST**						
Placebo contra Control	0.053	0.052	1.03	0.303	-0.048	0.155
Escitalopram contra Control	-0.106	0.043	-2.47	0.013	-0.189	-0.022

Supplementary Material

Behavioral intervention with task repetition compared to pharmacological intervention with SSRI for enhancement of cognitive control in emotional and non-emotional settings – Sklivanioti, Wang, Msghina

fNIRS MPFC

Table (Linear Mixed Models)

Fixed effects	
Stroop	CST [0] and EST [1]
Phase	Control [0] and Pharmacological Intervention [1]
Drug	Placebo [0] and Escitalopram [1]
Random effects	Subject effect. due to repeated measures

**Main effects and Interactions**	**Estimate**	**SE**	**z**	**P>|z|**	**95% CI (LL)**	**95% CI (UL)**
STROOP	0.085	0.061	1.40	0.160	-0.034	0.204
PHASE	0.015	0.060	0.25	0.801	-0.102	0.133
STROOP#PHASE	0.003	0.086	0.03	0.975	-0.165	0.171
DRUG	0.106	0.071	1.49	0.137	-0.034	0.245
STROOP#DRUG	-0.004	0.079	-0.05	0.960	-0.159	0.151
PHASE#DRUG	-0.110	0.078	-1.40	0.162	-0.263	0.044
STROOP#PHASE#DRUG	-0.010	0.111	-0.09	0.925	-0.228	0.207
Intercept	-0.102	0.054	-1.90	0.058	-0.207	0.003

Wald chi2(7) = 17.26

Prob > chi2 = 0.016

ICC = 0.41 (0.09 SE)

Behavioral intervention with task repetition compared to pharmacological intervention with SSRI for enhancement of cognitive control in emotional and non-emotional settings – Sklivanioti, Wang, Msghina

**Contrasts of interest**						
**Effects of pharmacological intervention**						
**CST**						
Placebo contra Control	0.015	0.060	0.25	0.801	-0.102	0.133
Escitalopram contra Control	-0.094	0.050	-1.88	0.061	-0.193	0.004
**EST**						
Placebo contra Control	0.018	0.061	0.29	0.771	-0.102	0.138
Escitalopram contra Control	-0.102	0.050	-2.03	0.043	-0.201	-0.003

fNIRS RPFC

Table (Linear Mixed Models)						
Fixed effects						
Stroop	CST [0] and EST [1]					
Phase	Control [0] and Pharmacological Intervention [1]					
Drug	Placebo [0] and Escitalopram [1]					
Random effects	Subject effect. due to repeated measures					
**Main effects and Interactions**	**Estimate**	**SE**	**z**	**P>|z|**	**95% CI (LL)**	**95% CI (UL)**

Behavioral intervention with task repetition compared to pharmacological intervention with SSRI for enhancement of cognitive control in emotional and non-emotional settings – Sklivanioti, Wang, Msghina

STROOP	0.001	0.048	0.02	0.986	-0.093	0.095
PHASE	-0.003	0.048	-0.06	0.950	-0.096	0.090
STROOP#PHASE	0.068	0.068	1.01	0.314	-0.065	0.202
DRUG	0.035	0.056	0.63	0.525	-0.074	0.144
STROOP#DRUG	0.053	0.063	0.84	0.400	-0.070	0.176
PHASE#DRUG	-0.088	0.062	-1.42	0.155	-0.210	0.033
STROOP#PHASE#DRUG	-0.063	0.088	-0.72	0.473	-0.236	0.110
Intercept	0.002	0.042	0.05	0.964	-0.080	0.084
Wald chi2(7) = 16.72						
Prob > chi2 = 0.019						
ICC = 0.39 (0.09 SE)						
**Contrasts of interest**						
**Effects of pharmacological intervention**						
**CST**						
Placebo contra Control	-0.003	0.048	-0.06	0.950	-0.096	0.090
Escitalopram contra Control	-0.091	0.040	-2.29	0.022	-0.170	-0.013
**EST**						
Placebo contra Control	0.065	0.049	1.35	0.178	-0.030	0.161
Escitalopram contra Control	-0.086	0.040	-2.16	0.031	-0.165	-0.008

Behavioral intervention with task repetition compared to pharmacological intervention with SSRI for enhancement of cognitive control in emotional and non-emotional settings – Sklivanioti, Wang, Msghina

2.2.2.2 Channel-wise

Cognitive Stroop Task							
**Escitalopram**	**Channel**	**t**	**p**	**Mean (Control Phase)**	**SD (Control Phase)**	**Mean (Intevention Phase)**	**SD (Intervention Phase)**
	1	1.206	0.242	0.068	0.219	0.019	0.190
	2	1.556	0.135	0.027	0.229	-0.046	0.182
	3	2.102	0.048	0.030	0.175	-0.041	0.180
	4	2.548	0.019	0.062	0.210	-0.048	0.174
	5	0.961	0.348	0.005	0.197	-0.033	0.223
	6	1.444	0.164	0.028	0.231	-0.034	0.193
	7	1.031	0.315	-0.052	0.253	-0.108	0.255
	8	1.258	0.223	0.019	0.252	-0.076	0.282
	9	1.364	0.188	-0.014	0.238	-0.071	0.254
	10	1.962	0.064	0.030	0.275	-0.126	0.276
	11	2.745	0.012	0.010	0.235	-0.083	0.229
	12	3.031	0.007	0.066	0.239	-0.059	0.183
	13	2.876	0.009	0.050	0.166	-0.044	0.183
	14	3.984	0.001	0.074	0.167	-0.049	0.178
	15	1.491	0.151	0.025	0.203	-0.017	0.162
	16	1.939	0.067	0.020	0.201	-0.051	0.146

Behavioral intervention with task repetition compared to pharmacological intervention with SSRI for enhancement of cognitive control in emotional and non-emotional settings – Sklivanioti, Wang, Msghina

**Placebo**	**Channel**						
	1	0.574	0.575	0.002	0.230	-0.030	0.178
	2	0.401	0.695	-0.031	0.196	-0.058	0.207
	3	-0.606	0.554	-0.072	0.173	-0.041	0.213
	4	-0.100	0.922	-0.069	0.217	-0.061	0.222
	5	-1.982	0.067	-0.197	0.197	-0.068	0.198
	6	-1.293	0.217	-0.114	0.168	-0.049	0.227
	7	-1.277	0.222	-0.179	0.198	-0.103	0.158
	8	-0.929	0.368	-0.182	0.213	-0.120	0.253
	9	-0.914	0.376	-0.081	0.161	-0.019	0.209
	10	1.813	0.091	-0.012	0.281	-0.159	0.341
	11	-1.414	0.179	-0.067	0.161	0.019	0.241
	12	-0.284	0.780	-0.053	0.189	-0.038	0.219
	13	-0.551	0.590	-0.006	0.116	0.017	0.168
	14	0.395	0.699	0.033	0.121	0.014	0.189
	15	0.610	0.552	-0.008	0.129	-0.035	0.147
	16	0.880	0.394	0.005	0.168	-0.049	0.187
**Emotional Stroop task**							
**Escitalopram**	**Channel**	**t**	**p**	**Mean (Control Phase)**	**SD (Control Phase)**	**Mean (Intevention Phase)**	**SD (Intervention Phase)**

	1	1.799	0.087	0.087	0.248	-0.020	0.208
	2	3.234	0.004	0.132	0.250	-0.035	0.172
	3	1.931	0.068	0.065	0.220	-0.033	0.188
	4	2.033	0.056	0.123	0.278	0.002	0.197
	5	1.628	0.119	0.092	0.282	-0.004	0.222
	6	1.956	0.065	0.098	0.267	0.002	0.206
	7	1.107	0.281	0.046	0.293	-0.020	0.222
	8	0.886	0.386	0.089	0.328	0.023	0.284
	9	2.074	0.051	0.072	0.264	-0.053	0.177
	10	2.410	0.026	0.116	0.303	-0.064	0.256
	11	1.892	0.073	0.082	0.276	-0.015	0.180
	12	2.460	0.023	0.134	0.280	<0.001	0.165
	13	1.695	0.106	0.071	0.232	-0.017	0.195
	14	1.521	0.144	0.079	0.218	<0.001	0.181
	15	1.381	0.182	0.087	0.239	0.017	0.177
	16	2.733	0.013	0.105	0.224	-0.028	0.177
**Placebo**	**Channel**						
	1	-0.918	0.375	0.035	0.242	0.098	0.198
	2	-0.615	0.549	-0.026	0.274	0.027	0.133
	3	-0.574	0.576	0.004	0.237	0.044	0.194
	4	-0.263	0.797	0.019	0.293	0.044	0.175
	5	-1.687	0.115	-0.057	0.216	0.067	0.208

	6	-0.765	0.458	-0.025	0.247	0.027	0.204
	7	0.145	0.887	0.001	0.281	-0.008	0.171
	8	-0.905	0.382	-0.104	0.338	-0.009	0.259
	9	-0.143	0.889	0.042	0.287	0.050	0.186
	10	-0.429	0.675	-0.119	0.265	-0.078	0.293
	11	-1.382	0.190	0.023	0.245	0.126	0.186
	12	0.136	0.894	0.070	0.285	0.059	0.184
	13	-0.760	0.461	0.019	0.191	0.073	0.188
	14	-0.497	0.627	0.048	0.210	0.079	0.164
	15	-1.066	0.306	0.001	0.218	0.072	0.179
	16	-1.774	0.100	-0.060	0.250	0.054	0.148

2.3 EDA

Behavioral intervention with task repetition compared to pharmacological intervention with SSRI for enhancement of cognitive control in emotional and non-emotional settings – Sklivanioti, Wang, Msghina

2.3.1 Variables

**Mean EDA Task**	Mean EDR frequency during all rest periods one session. CST or EST
**Event EDA Task**	Number EDR occurring during a block

2.3.2 Results

2.3.2.1 Mean EDA

**EDA Task** *Table (Linear Mixed Models)*	
Fixed effects	
Stroop	CST [0] and EST [1]
Phase	Control [0] and Pharmacological Intervention [1]
Drug	Placebo [0] and Escitalopram [1]
Random effects	Subject effect. due to repeated measures

**Main effects and Interactions**	**Estimate**	**SE**	**z**	**P>|z|**	**95% CI (LL)**	**95% CI (UL)**
STROOP	-0.007	0.019	-0.38	0.706	-0.044	0.030
PHASE	-0.002	0.018	-0.12	0.904	-0.038	0.034

Behavioral intervention with task repetition compared to pharmacological intervention with SSRI for enhancement of cognitive control in emotional and non-emotional settings – Sklivanioti, Wang, Msghina

STROOP#PHASE	-0.001	0.027	-0.03	0.974	-0.053	0.051
DRUG	0.011	0.026	0.42	0.674	-0.039	0.061
STROOP#DRUG	-0.015	0.025	-0.61	0.541	-0.065	0.034
PHASE#DRUG	0.005	0.025	0.20	0.845	-0.043	0.053
STROOP#PHASE#DRUG	0.011	0.035	0.31	0.759	-0.058	0.080
Intercept	0.129	0.019	6.87	<0.001	0.092	0.166
Wald chi2(7) = 3.36						
Prob > chi2 = 0.849						
ICC = 0.55(0.08 SE)						
**Contrasts of interest**						
**Effects of pharmacological intervention**						
**CST**						
Placebo contra Control	-0.002	0.018	-0.12	0.904	-0.038	0.034
Escitalopram contra Control	0.003	0.016	0.16	0.873	-0.030	0.035
**EST**						
Placebo contra Control	-0.003	0.019	-0.16	0.873	-0.041	0.035
Escitalopram contra Control	0.013	0.016	0.76	0.447	-0.020	0.045

Behavioral intervention with task repetition compared to pharmacological intervention with SSRI for enhancement of cognitive control in emotional and non-emotional settings – Sklivanioti, Wang, Msghina

2.3.2.2 Event-related analysis

Linear Mixed Models

**CST**	**coef**	**SE**	**z**	**p**	**95% CI (LL)**	**95% CI (UL)**
Control	-0.003	0.001	-2.295	0.022	-0.006	<0.001
Escitalopram	-0.006	0.002	-3.299	0.001	-0.010	-0.002
Placebo	-0.005	0.002	-2.160	0.031	-0.009	<0.001
EST						
Control	<0.001	0.002	-0.308	0.758	-0.003	0.003
Escitalopram	0.001	0.002	0.756	0.450	-0.002	0.005
Placebo	-0.002	0.003	-0.716	0.474	-0.007	0.003

2 Side-effects and blindedness

In the escitalopram arm, roughly 40% of subjects correctly guessed the identity of the given substance when asked 4 hours later. Roughly 13% of subjects in the placebo arm mistakenly thought they had received escitalopram, while the rest either thought they had received placebo or said they had no ground to hazard a guess. The most frequently reported side effects of single dose escitalopram were nausea (26%) and fatigue (11%) and of placebo fatigue (10%) followed by nausea (5%).

3 Power calculation

Supplementary Material

Behavioral intervention with task repetition compared to pharmacological intervention with SSRI for enhancement of cognitive control in emotional and non-emotional settings – Sklivanioti, Wang, Msghina

An a priori power analysis was conducted using Stata 14 software (StataCorp. 2015. Stata Statistical Software: Release 14. College Station. TX: StataCorp LP) to determine the minimum sample size required to test the study hypotheses. Results indicated the required sample size to achieve 80% power for detecting a medium effect (20% difference between control and intervention), at a significance criterion of $\alpha = .05$. was N = 20 for paired samples t-tests.
